# Independent Effects of Hypothyroidism and Obesity on Endometrial Cancer Risk Revealed by Mendelian Randomisation

**DOI:** 10.3390/biomedicines13071729

**Published:** 2025-07-15

**Authors:** Dylan M. Glubb, Xuemin Wang, Tracy A. O’Mara

**Affiliations:** Cancer Research Program, QIMR Berghofer Medical Research Institute, Brisbane, QLD 4006, Australia; dylan.glubb@qimrb.edu.au (D.M.G.); xuemin.wang@uq.net.au (X.W.)

**Keywords:** endometrial cancer, thyroid dysfunction, hypothyroidism, body mass index, genome-wide association study, Mendelian randomisation

## Abstract

**Objectives**: Thyroid dysfunction, particularly hypothyroidism, has been associated with endometrial cancer in observational studies; however, these findings may be confounded by obesity, an endometrial cancer risk factor. To clarify these associations, we performed Mendelian randomisation analysis, a genetic approach that mitigates confounding and reverse causation analyses. **Methods**: We accessed European-ancestry GWAS summary statistics for endometrial cancer (12,270 cases; 46,126 controls), endometrioid (8758 cases), and non-endometrioid (1230 cases) subtypes. Thyroid dysfunction phenotype and BMI GWAS data were predominantly from individuals of European descent. We used these datasets to conduct univariable and multivariable Mendelian randomisation analyses incorporating body mass index (BMI). **Results**: Our main finding was a causal association between hypothyroidism and decreased risk of endometrial cancer (OR = 0.93; 95% CI 0.89–0.97; *p* = 3.96 × 10^−4^). Subtype analysis revealed a decreased risk of the most common histological subtype, endometrioid endometrial cancer, and a similar protective association for Hashimoto’s thyroiditis, an autoimmune disease and common cause of hypothyroidism. Sensitivity analyses confirmed the robustness of the associations. Further analyses revealed that while BMI was causally associated with hypothyroidism risk, both BMI and hypothyroidism independently influenced endometrial cancer risk. **Conclusions**: Our study has identified hypothyroidism as a protective factor for endometrial cancer, challenging previous observational associations and highlighting potential confounding by obesity. Further investigation into immune mechanisms, particularly those linked to Hashimoto’s thyroiditis, may provide insights into the biological pathways underlying endometrial cancer development.

## 1. Introduction

Endometrial cancer is the most common gynaecological cancer in developed countries, with an increasing incidence that appears to be related to obesity [1]. Indeed, epidemiological studies have shown that obesity is one of the strongest risk factors for endometrial cancer [2]. To establish a causal relationship between obesity and endometrial cancer risk, Mendelian randomisation analyses have been conducted using germline genetic variants strongly associated with body mass index (BMI) as instrumental variables (IVs) [3]. By leveraging the random assortment of germline variants, which remain unaffected by disease or environmental factors, the Mendelian randomisation approach enables the assessment of causality while mitigating biases arising from confounding and reverse causation [4].

The thyroid gland is an important endocrine organ that, in response to thyroid stimulating hormone (TSH), produces two main hormones: triiodothyronine (T3) and thyroxine (T4). These hormones have multiple functions relevant to endometrial cancer development. For example, thyroid hormones regulate appetite, lipid storage, and metabolic rate [5], and they also affect the morphophysiology of the endometrium [6]. Thyroid dysfunction can manifest as hypothyroidism (high TSH levels and low T4 levels) or hyperthyroidism (low TSH levels and high T3 and T4 levels) and has been linked to endometrial cancer. For example, a prior diagnosis of thyroid disease was associated with increased endometrial cancer risk in a Danish record linkage study [7]. Furthermore, uterine cancer cases, primarily consisting of endometrial cancer, have been found to be more prevalent among Italian patients with thyroid disease compared to the general population [8]. Moreover, hypothyroidism is a common comorbidity for endometrial cancer [9], and consistent with this observation, increased TSH levels have been found in endometrial cancer patients in a small case–control study [10].

Another factor to consider in the relationship between thyroid dysfunction and endometrial cancer is obesity. Hypothyroidism and treatment of hyperthyroidism have been associated with weight gain or obesity [11]. However, Mendelian randomisation analyses have not provided evidence that alterations in levels of TSH, T3, or T4 affect obesity [12]. Intriguingly, both epidemiological and Mendelian randomisation studies suggest that obesity is a risk factor for thyroid dysfunction [12], particularly hypothyroidism [13,14].

Previous Mendelian randomisation analyses assessing the effects of thyroid dysfunction on uterine cancer have been limited by relatively small case numbers (n = 1931) and have yielded null results [15]. In the current study, we aimed to clarify these relationships using genetic data from a much larger number of endometrial cancer cases provided by the Endometrial Cancer Association Consortium (n = 12,270) [16]. We used Mendelian randomisation to evaluate the causal effects of multiple thyroid-related phenotypes, including hypothyroidism, hyperthyroidism, TSH, T3, and T4. In addition, we included autoimmune thyroid conditions (Hashimoto’s thyroiditis and Graves’ disease), as well as TPO antibody positivity, which is a biomarker of autoimmune thyroid disease [17]. Furthermore, given the role of obesity in the development of thyroid dysfunction and endometrial cancer, we have attempted to disentangle independent causal effects using multivariable Mendelian randomisation analysis. Together, these analyses aimed to clarify whether thyroid dysfunction causally influences endometrial cancer risk and whether this effect is independent of obesity.

## 2. Materials and Methods

### 2.1. Genome-Wide Association Study (GWAS) Summary Statistics

All data pertaining to human participants were obtained from publicly available studies, and analyses were conducted in accordance with institutional ethical standards. Details of all GWAS datasets used in this study—including trait, cohort ancestry, sample size, and whether summary statistics are sex-stratified—are provided in Supplementary Table S1. For endometrial cancer risk, data were derived from the largest GWAS meta-analysis of endometrial cancer risk [16]. To avoid potential bias from overlapping samples with other GWAS datasets used in the current study, UK Biobank samples were removed, resulting in 12,270 endometrial cancer cases and 46,126 controls for the generation of GWAS summary statistics [18]. For secondary analyses, we used summary statistics from GWAS of cases with either endometrioid (8758 cases and 46,126 controls) or non-endometrioid histology (1230 cases and 35,447 controls). Histological subtypes of endometrial cancer were confirmed based on pathology reports, as described previously [16,19].

For autoimmune thyroid disease, we used GWAS summary statistics from UK Biobank and Iceland cases and controls [20]. GWAS summary statistics for hypothyroidism were derived from a meta-analysis of cases and controls of European ancestry from UK Biobank and FinnGen [21]. GWAS summary statistics for hyperthyroidism were obtained from a meta-analysis of cases and controls with European ancestry from 19 cohorts [22]. For Graves’ disease and Hashimoto’s thyroiditis, GWAS had been performed using participants with European ancestry but included only 2285 and 462 cases, respectively [23]. As these GWAS had limited power to detect meaningful associations for these diseases, we used GWAS summary statistics from meta-analyses of two European and one East Asian population [24]. For Graves’ disease, 1678 cases from UK Biobank and FinnGen (release 3) were meta-analysed with 2809 cases from Biobank Japan; for Hashimoto’s thyroiditis, 15,654 cases from UK Biobank and FinnGen were meta-analysed with 537 cases from Biobank Japan.

In addition to disease-specific GWAS, we also used summary statistics for thyroid hormones or markers. GWAS for free T3 levels had been performed in disease-free participants from three Croatian cohorts, but full summary statistics were not available [25]. For TPO antibody positivity, we used data from a two-stage GWAS of 11 European populations, but full summary statistics were also not available [26]. For both free T4 and TSH levels, female-stratified and sex-combined GWAS summary statistics were used. For female-stratified and sex-combined free T4, GWAS summary statistics were derived from a meta-analysis of 26 cohorts [22]. For female TSH levels, summary statistics were available from a GWAS meta-analysis of women from 30 cohorts [22], and for sex-combined TSH levels, we used summary statistics from a GWAS meta-analysis of three studies [27]. For BMI, we used GWAS summary statistics derived from 434,794 women of European ancestry in a meta-analysis of the Genetic Investigation of Anthropometric Traits consortium and UK Biobank [28].

### 2.2. IV Selection

GWAS summary statistics from all phenotypes were used to identify genetic variants associated with exposures at genome-wide levels (*p* < 5 × 10^−8^); no such variants were available for non-endometrioid endometrial cancer. To identify independent IVs, we used a window of 10 Mb and maximal linkage disequilibrium of r^2^ = 0.001 between instruments in PLINK [29]. The linkage disequilibrium reference used a random sample of 10,000 unrelated participants from the UK Biobank [18]. Palindromic variants (i.e., those with A/T or G/C alleles) with a minor allele frequency ≥0.42 were excluded to prevent errors due to strand ambiguity. Variants from the *SH2B3* locus were also excluded, as this is a pleiotropic locus and, through GWAS, has been associated with many traits including hypothyroidism [30], Hashimoto’s thyroiditis [24], and endometrial cancer [16]. To assess weak instrument bias, we calculated the *F*-statistic [31] for each instrumental variable and all IVs of an exposure using the formula *F* = R^2^ × (N − 1 − k)/((1 − R^2^) × k), where R^2^ is the proportion of variance explained by the instrumental variable, N is the sample size, and k is the number of IVs included in the analysis [32]. For the calculation of *F*-statistic for each instrumental variable, k equals 1. The proportion of variance explained by IVs was calculated using the formula in the Supplementary Methods [32]. An *F*-statistic > 10 is often used to indicate sufficient instrument strength. For quantitative traits, such as circulating thyroid hormones, IVs are reported as the association with one standard deviation increase in the levels of the respective hormone. Details of the IVs used in Mendelian randomisation analyses can be found in Supplementary Table S2.

### 2.3. Mendelian Randomisation Analysis

To assess causal relationships, we used the two-sample Mendelian randomisation framework [33], following the Strengthening the Reporting of Observational Studies in Epidemiology using the Mendelian Randomisation checklist (Supplementary Information) [34,35]. For free T3, the only exposure with a single IV, we used the Wald ratio to estimate effects. For all other exposures, multiple IVs were combined using the inverse-variance weighted (IVW) random effect method as the primary analysis. The IVW method uses all available IVs and thus has the most power to detect an association, assuming that the IVs meet certain criteria: (1) they are reliably associated with the exposure of interest; (2) they are not associated with any confounders that mediate the association between exposure and outcome; and (3) they only affect the outcome through the exposure, without any horizontal pleiotropy. To detect violations in the underlying Mendelian randomisation assumptions, we assessed heterogeneity in the causal effect estimates across IVs by calculating Cochran’s Q statistic and its associated *p*-value [36]. Wald’s ratio, IVW, and Cochran’s Q statistic analyses were implemented using the TwoSampleMR (version 0.5.6) R package [37].

We used the online Mendelian randomisation power calculator mRnd (https://shiny.cnsgenomics.com/mRnd/, accessed on 6 March 2023) [38] to calculate the power for Mendelian randomisation analyses to detect an association between one standard deviation change in the trait of interest under scenarios reflecting weak, moderate, and strong effects with cut-offs set to OR > 1.1, OR > 1.2, and OR > 1.4, respectively. For the calculation of power for continuous outcomes, variances of the exposure and outcome variables were both set to 1 given that the GWASs for outcome were conducted on the inverse-normalised variable. The results of the power calculations can be found in Supplementary Table S3.

To determine the effects of thyroid dysfunction phenotypes on endometrial cancer risk, we used a Bonferroni correction threshold, adjusting for the testing of 11 traits (*p* < 4.5 × 10^−3^). We also performed bidirectional analyses, using thyroid dysfunction phenotypes as outcomes and risk of endometrial cancer (and its histological subtypes) as exposures. To investigate the involvement of BMI in potential causal relationships, we conducted bidirectional MR analysis between BMI and exposures that were associated with endometrial cancer risk. We were unable to study free T3 or TPO antibody positivity as outcomes due to the unavailability of full GWAS summary statistics for these phenotypes. We were also unable to examine the effects of non-endometrioid endometrial cancer risk due to the absence of IVs for this trait.

### 2.4. Mendelian Randomisation Sensitivity Analyses

IV assumptions (2) and (3) are often violated in a Mendelian randomisation setting. Therefore, to account for potential violations of these assumptions and evaluate the robustness of associations identified by IVW analysis, we conducted sensitivity analyses using the MR-Egger, weighted median, and the Mendelian randomisation pleiotropy residual sum and outlier (MR-PRESSO) methods. The MR-Egger method relaxes assumption (2) by allowing a non-zero regression intercept [39]. This provides an unbiased causal estimate even when assumption (2) is violated for all IVs, as long as the magnitude of the horizontal pleiotropic effects is independent of the variant-exposure effects. The MR-Egger method also provides a formal test of the existence of horizontal pleiotropy [39]. The weighted median method assumes at least half of the variants are valid IVs and so provides a reliable estimate even if the assumptions for some IVs are violated [40]. Lastly, MR-PRESSO identifies and adjusts for outlier IVs displaying horizontal pleiotropic effects [41]. As these sensitivity analyses do not yield reliable results when using small numbers of IVs, they were only performed when a minimum of eight IVs were available. The MR-Egger and weighted median methods were implemented in the TwoSampleMR (version 0.5.6) R package [37]; additionally, we used the MR-PRESSO (version 1.0) R package. A threshold of *p* < 0.05 was applied for sensitivity analyses.

### 2.5. Multivariable Mendelian Randomisation Analysis

To investigate the independent effects of BMI and hypothyroidism on endometrial cancer risk, we conducted multivariable Mendelian randomisation analysis. We first clumped genetic variants with GWAS summary statistics for BMI and hypothyroidism together in PLINK using a window of 10 Mb and a maximal linkage disequilibrium of r^2^ = 0.001 between variants, as per the univariable Mendelian randomisation analyses. As there are far more genetic variants associated with BMI, we preferentially selected variants for hypothyroidism that were in linkage disequilibrium with variants associated with BMI to maximise the number of IVs for hypothyroidism. A list of the selected IVs is provided in Supplementary Table S4. The multivariable analysis was performed using the MendelianRandomisation (version 0.6.0) R package [42]. R code for the multivariable analysis can be found in the Supplementary Information. To confirm the robustness of the results from the multivariable analysis, we also reran univariable Mendelian randomisation analyses with endometrial cancer risk as the outcome using only IVs included the multivariable analysis for BMI or hypothyroidism risk.

## 3. Results

### 3.1. Hypothyroidism Is Causally Associated with Decreased Endometrial Cancer Risk

**Figure 1:**
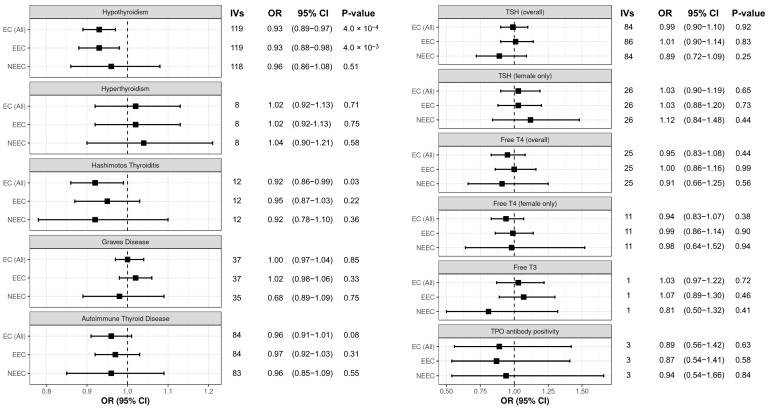
Each panel displays the IVW analysis results for the associations between thyroid dysfunction phenotypes (as indicated) and the risk of all endometrial cancer (EC (All)), endometrioid endometrial cancer (EEC), and non-endometrioid endometrial cancer (NEEC). Numbers of IVs used in each analysis are shown.

Mendelian randomisation analysis revealed a causal association between hypothyroidism and decreased endometrial cancer risk (OR = 0.93; 95% CI 0.89–0.97; *p* = 3.96 × 10^−4^; Figure 1). Although no other thyroid dysfunction phenotype passed the Bonferroni multiple correction threshold for association with endometrial cancer risk (*p* < 4.5 × 10^−3^; Figure 1), we did observe a nominal association between Hashimoto’s thyroiditis and decreased endometrial cancer risk (OR = 0.92, 95% CI 0.86–0.99; *p* = 0.03; Figure 1). As Hashimoto’s thyroiditis accounts for most cases of hypothyroidism in developed countries [43], the two findings are consistent with each other. In the analysis of endometrial cancer histological subtypes, we also found a causal association between hypothyroidism and decreased endometrioid endometrial cancer risk (OR = 0.93, 95% CI 0.88–0.98; *p* = 4.02 × 10^−3^; Figure 1). However, there was some evidence of IV heterogeneity in this analysis from Cochran’s Q statistic (*p* < 0.05; Supplementary Table S5). Bidirectional Mendelian randomisation analysis did not support effects of endometrial cancer risk on thyroid dysfunction phenotypes (Supplementary Table S6).

### 3.2. Sensitivity Analysis of Associations Between Thyroid Dysfunction and Endometrial Cancer Risk

**Figure 2:**
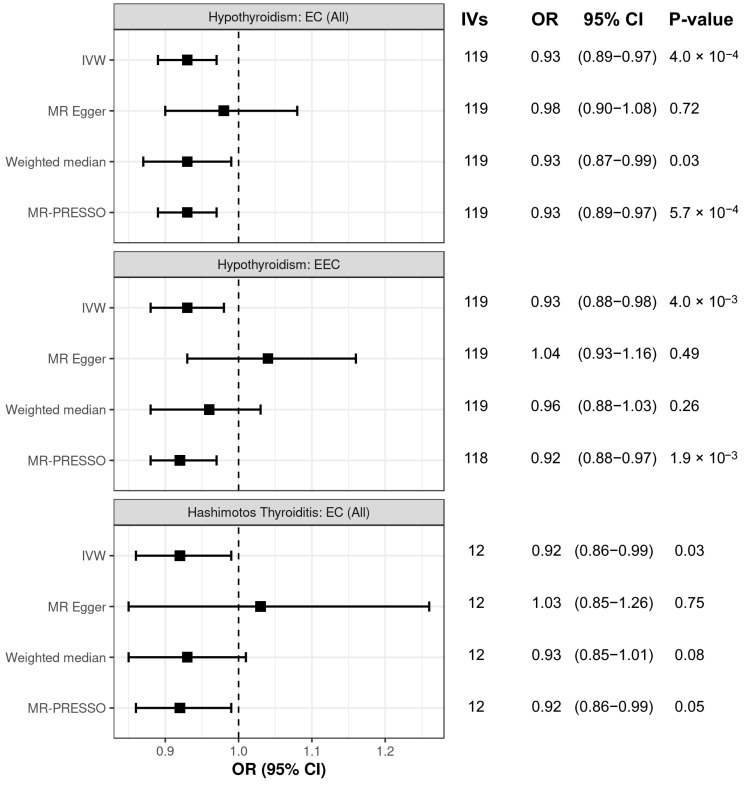
Sensitivity analysis of the association between hypothyroidism and risk of all endometrial cancer (EC (All)), hypothyroidism and risk of endometrioid endometrial cancer (EEC), and between Hashimoto’s thyroiditis and risk of EC (All). Panels display sensitivity analyses using MR-Egger, weighted median, and MR-PRESSO approaches, and the primary IVW analysis. Number of IVs used in each analysis is shown.

We performed sensitivity analyses to evaluate the associations of hypothyroidism and Hashimoto’s disease with endometrial cancer risk. These analyses supported the association between hypothyroidism and decreased risk of endometrial cancer, as demonstrated by associations in the weighted median and MR-PRESSO analyses (Figure 2). For the association of hypothyroidism with endometrioid endometrial cancer risk, the MR-Egger analysis showed a discordant direction of effect (Figure 2) and suggested the presence of horizontal pleiotropy (Supplementary Table S5). However, by removing an IV that contributed to heterogeneity (Supplementary Table S5), the MR-PRESSO analysis demonstrated an association between hypothyroidism and decreased endometrioid endometrial cancer risk, consistent with the IVW analysis (Figure 2). Lastly, the MR-Egger analysis again provided a discordant direction of effect for the association of Hashimoto’s thyroiditis with endometrial cancer risk (Figure 2), but there was no evidence of pleiotropy (Supplementary Table S5). Furthermore, the directionality of the MR-PRESSO and weighted median analyses were consistent with the association of Hashimoto’s thyroiditis with reduced endometrial cancer risk (Figure 2).

### 3.3. BMI and Hypothyroidism Are Independently Associated with Endometrial Cancer Risk

**Figure 3:**
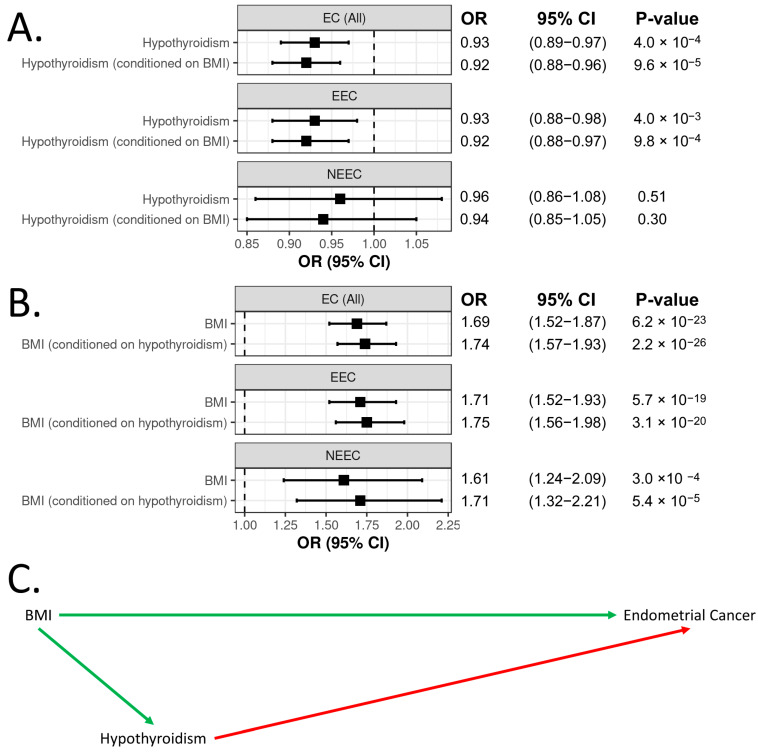
Panels (**A**,**B**) show the univariable or multivariable associations of BMI and hypothyroidism, respectively, with risk of all endometrial cancers (EC (All)), endometrioid endometrial cancer (EEC), and non-endometrioid endometrial cancer (NEEC). The relationships between BMI, hypothyroidism, and endometrial cancer are depicted in a directed acyclic graph (**C**). Positive associations are indicated by green arrows, while negative associations are shown by red arrows.

To assess the effect of BMI on the relationship between hypothyroidism and endometrial cancer risk, we firstly performed bidirectional Mendelian randomisation analysis of BMI and hypothyroidism. Our findings revealed that increased BMI was very strongly associated with increased hypothyroidism risk, but there was no evidence for an effect of predisposition to hypothyroidism on BMI (Supplementary Table S7). Although some IV heterogeneity was detected in the IVW analysis of BMI’s effect on hypothyroidism, the finding remained in all sensitivity analyses (Supplementary Table S7). We next performed multivariable Mendelian randomisation, which demonstrated that both BMI and hypothyroidism were independently associated with endometrial cancer risk, with BMI strongly increasing risk and hypothyroidism modestly decreasing risk (Figure 3A,B). Similar associations were also observed for endometrioid endometrial cancer risk (Figure 3A,B). Moreover, the multivariable analyses showed slightly stronger associations compared to the univariable analyses, particularly for the effect of BMI on endometrial cancer risk, including the histological subtypes (Figure 3B). These observations suggest that hypothyroidism acts as a mediator in the causal pathway between BMI and endometrial cancer (Figure 3C). Specifically, increased BMI raises the risk of hypothyroidism, which in turn has a protective effect on endometrial cancer.

## 4. Discussion

In contrast to previous observational studies, Mendelian randomisation analysis revealed a causal association between hypothyroidism and decreased endometrial cancer risk. Additionally, we found a suggestive association between Hashimoto’s disease and reduced endometrial cancer risk. This finding indicates that the effect of hypothyroidism on endometrial cancer risk may be driven by Hashimoto’s thyroiditis, the leading cause of hypothyroidism in developed countries. Subtype analysis uncovered a causal association between hypothyroidism and decreased risk of endometrioid endometrial cancer, which constitutes the majority of endometrial cancer cases both in the general population and the endometrial cancer GWAS dataset. Sensitivity analyses demonstrated the robustness of the associations of hypothyroidism with endometrial cancer risk and provided support for the potential effect of Hashimoto’s thyroiditis.

We did not detect any associations between thyroid hormone levels and endometrial cancer risk in our analysis. This contrasts with a previous Mendelian randomisation study by Yuan et al., which reported a nominal association between TSH levels and uterine cancer risk [15]. Yuan et al. also studied 21 other site-specific cancers and found that TSH levels were associated with reduced risk of overall cancer. Despite having sufficient statistical power to detect weak to moderate effects of TSH levels, we found no evidence of such an association with endometrial cancer risk.

The mechanism underlying the protective effect of hypothyroidism remains unclear but may involve thyroid autoimmunity or related immune–endocrine pathways. For example, hypothyroidism often develops in endometrial cancer patients as a result of immune activation therapy [44,45], suggesting that the effect of hypothyroidism on endometrial cancer may be driven by Hashimoto’s disease. Systemic lupus erythematosus (SLE), another autoimmune disease, has been robustly associated with reduced endometrial cancer risk in Mendelian randomisation analyses [46]. Furthermore, Mendelian randomisation analysis indicates that there is a bidirectional relationship between hypothyroidism and SLE, with a particularly strong effect of hypothyroidism on increased SLE risk [47]. The protective effect of SLE on endometrial cancer has been proposed to be related to antinuclear autoantibodies that target cancer cells with defective DNA repair [46]. Thyroid autoantibodies may have a similar effect. Indeed, an observational study found that uterine cancer was less prevalent in thyroid disease patients who had thyroid autoantibodies compared to thyroid disease patients who tested negative for autoantibodies [8].

To evaluate the impact of BMI on the relationship between hypothyroidism and endometrial cancer risk, we conducted additional Mendelian randomisation analyses. Bidirectional analysis revealed a strong causal association between increased BMI and hypothyroidism risk, while there was no effect of hypothyroidism on BMI. In multivariable analyses, both BMI and hypothyroidism demonstrated independent effects on endometrial cancer (including the endometrioid subtype): hypothyroidism remained associated with decreased risk, while BMI was associated with increased risk. Notably, we found that adjusting for hypothyroidism slightly strengthened the association between BMI and endometrial cancer risk. Thus, hypothyroidism appears be a mediator of the relationship, attenuating the effect of BMI on endometrial cancer risk. Moreover, this finding provides an explanation for the association of hypothyroidism with endometrial cancer in observational studies. Specifically, as obesity is an endometrial cancer risk factor and is more prevalent among cases, it follows that the prevalence of hypothyroidism will also be increased in these individuals.

### Limitations

Several limitations should be acknowledged. Firstly, the statistical power of our analyses varied across thyroid dysfunction phenotypes, with limited power to detect low to moderate effects of phenotypes such as hyperthyroidism, Graves’ disease, and TPO antibody positivity. As a result, the lack of associations for these phenotypes could be the result of insufficient statistical power rather than a true absence of association. This limitation highlights the need for larger GWAS datasets and additional IVs to capture more of the trait variance for future analyses. Secondly, the analyses of the non-endometrioid subtype were exploratory in nature and should also be interpreted with caution. There was low statistical power to detect associations with this outcome, and heterogeneity exists within the non-endometrioid subtype, which encompasses several different histologies. Future GWASs with larger sample sizes and stratification of non-endometrioid cases are warranted to better understand the associations within this subtype. Finally, the generalisability of our findings may be limited to populations of European ancestry, as the genetic data used in our study predominantly represented individuals from this genetic background. To validate and generalise these findings, it is crucial for future studies to encompass more diverse populations.

## 5. Conclusions

Our study establishes a robust causal association between hypothyroidism and a decreased risk of endometrial cancer. However, the underlying mechanism for the potential protective effect of hypothyroidism remains unclear and warrants further investigation, with a potential role for autoimmune responses. Additional analyses revealed that BMI is causally associated with hypothyroidism risk, with both factors independently influencing endometrial cancer risk.

## Data Availability

All data analysed in this study are publicly available, and their sources have been referenced throughout the manuscript and Supplementary Materials.

## Supplementary Materials

The following supporting information can be downloaded at: https://www.mdpi.com/article/10.3390/biomedicines13071729/s1, Table S1: study information; Table S2: IVs used in Mendelian randomisation analyses; Table S3: Mendelian randomisation power calculations; Table S4: IVs included in the multivariable Mendelian randomisation analysis; Table S5: Mendelian randomisation sensitivity analyses; Table S6: Effect of endometrial cancer risk on thyroid dysfunction phenotypes in Mendelian randomisation analysis; Table S7: Bidirectional MR analysis of hypothyroidism and BMI. Supplementary information: STROBE-MR checklist.

## Abbreviations

The following abbreviations are used in this manuscript:

BMI	Body mass index
CI	Confidence interval
EEC	Endometrioid endometrial cancer
EC	Endometrial cancer
GWAS	Genome-wide association study
IV	Instrumental variable
IVW	Inverse-variance weighted
NEEC	Non-endometrioid endometrial cancer
OR	Odds ration
SLE	Systemic lupus erythematosus
T3	Triiodothyronine
T4	Thyroxine
TPO	Thyroid peroxidase
TSH	Thyroid stimulating hormone
